# Age-related differences in working hours among male and female GPs: an SMS-based time use study

**DOI:** 10.1186/s12960-017-0258-4

**Published:** 2017-12-19

**Authors:** Daniël van Hassel, Lud van der Velden, Dinny de Bakker, Ronald Batenburg

**Affiliations:** 1 NIVEL, the Netherlands Institute for Health Services Research, P.O. Box 1568, 3500 BN Utrecht, The Netherlands; 2 CAOP, P.O. Box 556, 2501 CN Den Haag, The Netherlands; 3 Tranzo, Scientific Centre for Transformation in Care and Welfare, Tilburg University, P.O. Box 90153, 5000 LE Tilburg, The Netherlands; 4 Department of Sociology, Radboud University Nijmegen, P.O. Box 9104, 6500 HE Nijmegen, The Netherlands

**Keywords:** General practitioners, Health workforce planning, Path analysis, Working hours

## Abstract

**Background:**

In several countries, the number of hours worked by general practitioners (GPs) has decreased, raising concern about current and impending workforce shortages. This shorter working week has been ascribed both to the feminisation of the workforce and to a younger generation of GPs who prefer more flexible working arrangements. There is, however, limited insight into how the impact of these determinants interact. We investigated the relative importance of differences in GPs’ working hours in relation to gender, age, and employment position.

**Methods:**

An analysis was performed on real-time monitoring data collected by sending SMS text messages to 1051 Dutch GPs, who participated during a 1-week time use study. We used descriptive statistics, independent sample *t*-tests, and one-way ANOVA analysis to compare the working time of different GP groups. A path analysis was conducted to examine the difference in working time by gender, age, employment position, and their combinations.

**Results:**

Female GPs worked significantly fewer hours than their male peers. GPs in their 50s worked the highest number of hours, followed by GPs age 60 and older. GPs younger than 40 worked the lowest number of hours. This relationship between working hours and age was not significantly different for women and men. As shown by path analysis, female GPs consistently worked fewer hours than their male counterparts, regardless of their age and employment position. The relationship between age and working hours was largely influenced by gender and employment position.

**Conclusions:**

The variation in working hours among GPs can be explained by the combination of gender, age, and employment position. Gender appears to be the most important predictor as the largest part of the variation in working hours is explained by a direct effect of this variable. It has previously been reported that the difference in working hours between male and female GPs had decreased over time. However, our findings suggest that gender remains a critical factor for variation in time use and for policy instruments such as health workforce planning.

## Background

Many countries fear, or are already confronted with, shortages of general practitioners (GPs) [1–3]. This is not only caused by an increasing demand for primary care, but also by the decreasing number of hours worked by GPs [4, 5]. This development is often ascribed to changes in the composition of the GP labour supply and to their personal preferences. An important change is the steady feminisation of the profession seen in the Netherlands and in other European countries [6]. In the period 2005–2015, the share of Dutch female GPs has increased from 33 to 48% [7]. This percentage will continue to increase in the coming years as most of the trainees are women [8–10].

One of the consequences of this feminisation for the GP workforce is that female GPs are more likely to work part-time or take career breaks than their male counterparts [11, 12]. Female GPs prefer more flexible working positions and serve fewer patients [13, 14], which has led to concerns about the availability and accessibility of GP care [15]. The number of working hours not only differs between the sexes but also depends upon the life course of GPs. Several studies have showed that women, especially at younger ages, work fewer hours than their male counterparts [14, 15]. For example, a time use survey conducted in the UK revealed that female GPs worked 11 h fewer than male GPs, because women chose to invest their time in the care of their children [16]. The number of hours women work can be understood as the result of how they choose to balance their time between family and work [17]. This assumes ‘freedom of choice’, but as sociologists and gender scholars pointed out, a number of constraints determine this choice of behaviour. A key constraint on the preferred working time of women is the idea and discourse of gender roles in which domestic duties are considered a woman’s work, not only by men, but also by women [18]. Indeed, numerous studies have shown that women spend considerably more time on child care, and on domestic tasks in general, and less on paid jobs [19].

There are, however, new developments in the division of domestic tasks between men and women in different professions, and among GPs in particular. In recent years, male employees also spent more time on family duties and have a greater need for flexible working hours. As a result of this, the differences in working hours between male and female GPs have become smaller over the course of time. There is a new generation of GPs who tend to work fewer hours [6].

A shortcoming of the existing studies on gender and the working hours of GPs, however, is that these provide limited insight into how gender differences in working time are influenced by other factors such as age and employment positions. In addition, the effect of how these factors interact on GPs’ working time is also important. While age is a proxy for the family duties GPs are expected to embrace, one’s employment position is important as women especially, and younger GPs too, prefer to work on a salaried basis. This enables them to choose a shorter and more flexible working week and is more compatible with childcare commitments [20–22]. Another limitation of previous studies is the measurement of working time. Different types of survey and diary data raise question marks about their validity. In this paper, we use and analyse new and more valid data on GPs’ working time.

The aim of the present paper is to investigate the relative impact of gender and age on the working hours of Dutch GPs by applying a path analysis model. We will also provide insight into the effects of how these variables interact, in particular with regard to the employment position of GPs. The analyses are based on real-time data of GPs’ working hours collected by a large Dutch time use study.

## Methods

### Data collection

The analyses in this paper are based on time use data collected in order to estimate the working hours of GPs per week as precisely as possible. The time use survey was not set up in a traditional manner, for weekly monitoring was conducted by means of an SMS application. Messages were sent randomly during one full diary week to each GP participating in the study. GPs were texted every day to measure their activities during time slots of 3 h. Exceptions were made when they indicated that they would be out of their office during a part of the day. The text messages asked GPs to select one of four exclusive answers in reply to an SMS message. These were as follows: “At this moment I am; (a) not working as a GP; (b) working directly with patients; (c) working indirectly with patients, or; (d) working as a GP but not directly or indirectly with patients”. Fifty-six messages were scheduled per GP, per week. The data collection was conducted in 57 consecutive weeks from December 2012 to January 2014.

During the period of field work, more than 5000 letters of invitation were sent in two monthly batches in order to ensure a sufficient number of GPs were recruited. Seven stratified samples by employment position and gender were drawn from the NIVEL national registration of GPs [7]. In addition to the letters of invitation, media announcements were made in several newsletters and websites in order to encourage GPs to sign up for the study. In total, 1051 GPs in both full, and part-time employment, participated in the period studied. This was, on average, 19 GPs per week, with 44 GPs participating twice. The study resulted in 61,320 time data point measurements. 

**Table:** Box 1 Calculating the working hours based on SMS

Working hours were calculated by multiplying the replies to the questions about activities by three as these were the time slots in which the messages were sent during the week. A GP who replied 13 times one of the answers, b, c, or d (“At this moment I am working; (b) directly; (c) indirectly, or; (d) not directly or indirectly with patients”) would work 13 × 3 = 39 h. This provides a broad estimate of every GP’s working week. However, the method is appropriate when more participants are included, because this results in an increasing number of measurements for a target group as a whole. An accurate calculation of the average working hours can then be made.	

All groups of GPs, based on gender and employment position, were represented sufficiently in most of the SMS weeks, providing power to execute split sampling and subgroup analyses. More detailed information of the SMS instrument is described elsewhere [23].

### Data analysis

With regard to the employment position of GPs, our research sample contained self-employed GPs, salaried GPs who work in service of another GP, and GP locums. We focus here for practical reasons on the first two groups. In doing so, both employment position and gender were able to be coded as dummy variables. The results below are therefore based on a selection of the time use data collected—that is, the monitoring data from 856 GPs.

We first compared means and standard deviations of the working hours of different groups of GPs based on their gender, age and employment position. Then bivariate analyses were performed depending on the type of variables as defined by independent samples *t*-tests or one-way ANOVA (*f*-tests). For the next analyses, we excluded the GPs above the age of 60 to exclude the effect of early retirement on working hours. A multiple linear regression analysis was performed with the independent variables representing a GP’s gender and age and working hours as the dependent variable. Both unstandardised (b) and standardised (beta) regression coefficients were calculated to measure the effect size and the relative effect of the independent variables on working hours. Then, we added the interaction term of gender and age to analyse to what extent these two variables have an interacting effect on working hours. Finally, a path analysis was conducted to examine the relationships between gender, age, and employment position as predictors of working hours. To explore the relationships between these variables, we computed both Pearson correlations and standardised regression coefficients, thus disentangling the direct and indirect relationships between them. The tolerance/variance inflation factor (VIF) was calculated to check for multicollinearity between the independent variables age, gender, and employment position on working hours. This proved that there was no need to remove one of the variables from the analyses. The statistical analyses were performed in Stata 14.0.

## Results

### Mean hours worked

Taking the self-employed and salaried GPs together, the GPs in our study reported working 44.9 h per week on average (Table 1). There are significant differences between male and female GPs and between GPs of different age groups. Male GPs work, on average, 8 h more than female GPs. Concerning age, it is shown that GPs in their 50s worked the highest number of hours, followed by GPs age 60 or older. GPs younger than 40 worked the lowest number of hours. Table 1 also shows that there is a relatively large and significant difference in working hours between self-employed GPs (48.4) and salaried GPs (34.5).

**Table 1:** Number of GPs and mean number of working hours per week, divided by gender, age, and employment position

	*N*	(%)	Mean hours	(sd)	
Total	856	(100.0)	44.9	(15.3)	
Gender					***
Male	364	(42.5)	49.5	(15.8)	
Female	492	(57.5)	41.5	(14.0)	
Age					***
< 40	293	(34.2)	39.7	(13.5)	
40–49	257	(30.0)	45.0	(14.4)	
50–59	259	(30.3)	50.3	(16.0)	
≥ 60	47	(5.5)	47.9	(16.2)	
Employment position					***
Self-employed	642	(75.0)	48.4	(14.8)	
Salaried	214	(25.0)	34.5	(11.6)	

***p < 0.01

### Multiple regression analysis: the interacting effect of gender and age on working hours

The first linear regression equation estimates the independent effect of age and gender on the working hours of GPs (Table 2, model 1). In accordance with Table 1, the differences in the working hours of GPs, by gender and age, remain significant. An effect is found for gender (female GPs work fewer hours compared to their male counterparts (*β* = − 0.216, *b* = − 6.726)) and a positive effect of age (*β* = 0.243, *b* = 0.433).

**Table 2:** Effect of gender and age on the number of working hours for GPs under the age of 60 (multiple regression)

	Model 1				Model 2			
	B	Beta	*p* value		B	Beta	*p* value	
Intercept	29.700				33.062			
Gender (male=ref)	− 6.726	− 0.216	0.000	***	− 12.731	− 0.409	0.020	**
Age	0.433	0.243	0.000	***	0.360	0.203	0.000	***
Gender*age					0.135	0.193	0.262	
Adjusted *R* square		12.5%	0.000	***		12.6%	0.000	***

**p < 0.05; ***p < 0.01

**Fig. 1:**
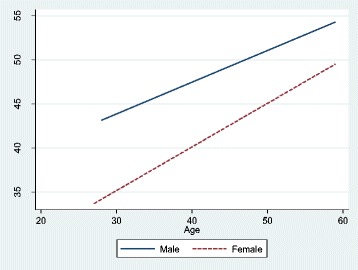
Effects of age on working hours for male and female GPs under the age of 60

When the interaction term between gender and age is added to the model (model 2), it appears that the main effect of gender is considerably higher (*β* = − 0.409, *b* = − 12.731) while the main effect of age (*β* = 0.203, *b* = 0.360) is lower compared to model 1. There is, however, no significant interaction effect between age and gender. This confirms that the relationship between the number of working hours and age does not significantly differ between women and men. This is also illustrated in Fig. 1.

### Path analysis: causal relationships between gender, age, employment position, and working hours

#### Correlations

The Pearson correlation in Table 3 reveals the relationships presented above between gender, age, employment position, and working hours. Firstly, there is a negative linear correlation between gender and working hours (*r* = − 0.266) confirming that women generally work fewer hours than men. Secondly, there is a positive correlation between age and working hours (*r* = 0.288) showing that the average number of working hours is higher for the older age categories of GPs.

**Table 3:** Pearson correlations of gender, age, employment position, and number of working hours for GPs under the age of 60

	Gender		Age		Employment position		Working hours
Gender (0=male, 1=female)	1.000						
Age	− 0.204	***	1.000				
Employment position (0=self-employed, 1=salaried)	0.144	***	− 0.459	***	1.000		
Working hours	− 0.266	***	0.288	***	− 0.400	***	1.000

***p < 0.01

#### Path analysis model

**Fig. 2:**
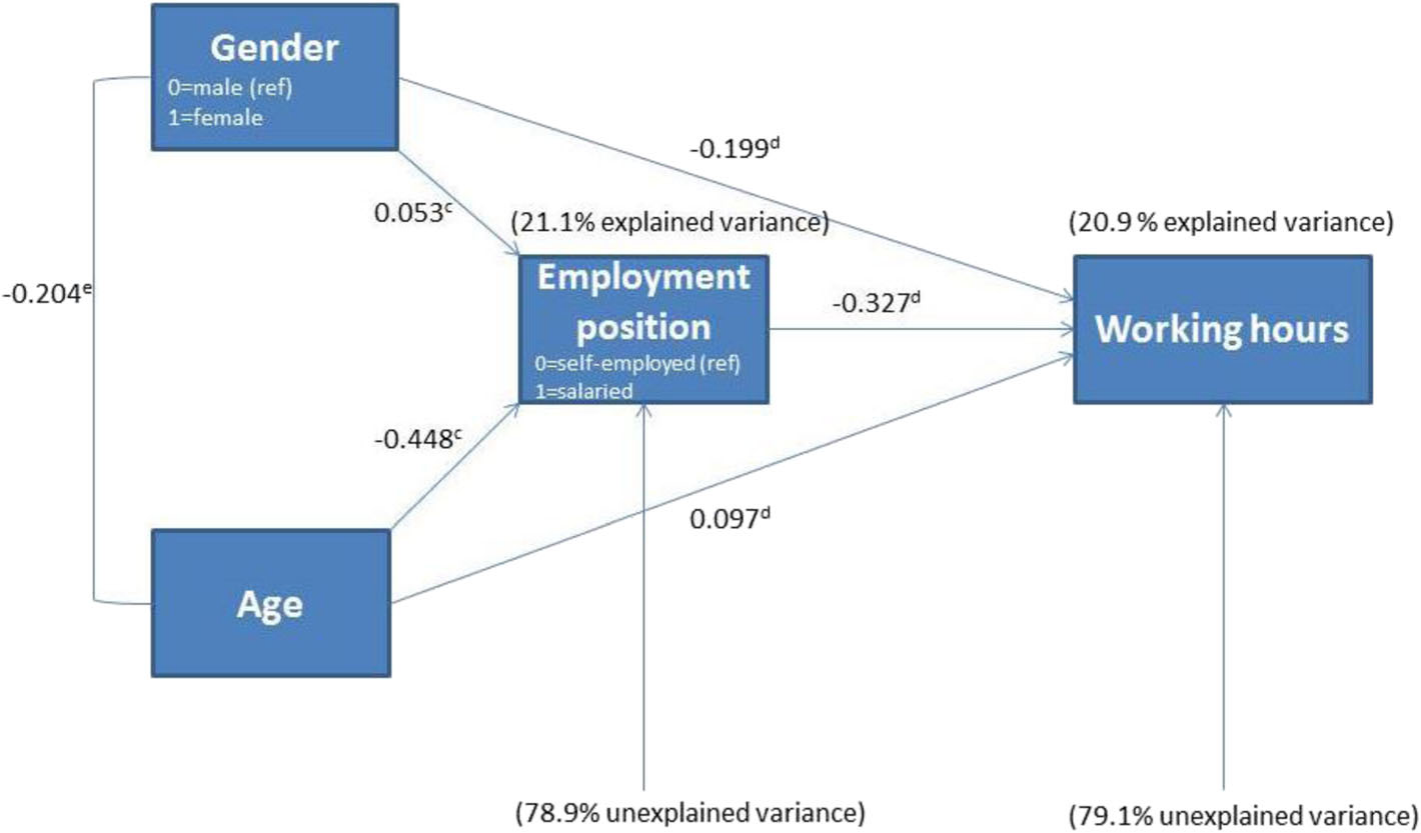
Effects of gender and age on working hours of GPs under age 60*.* The relationship between gender and age is the correlation coefficient of Table 3, because this relationship goes both ways. Effect of gender on employment position is significant at 90% confidence level. All other results are significant at 99% confidence level. (c) Beta-coefficient of Table 5 in Appendix. (d) Beta-coefficient of Table 6 in Appendix. (e) Correlation-coefficient of Table 3

To disentangle the direct and indirect relationships behind the bivariate correlations in Table 3, two additional multiple regression analyses were performed. These were firstly, taking the employment position and, secondly, taking working hours, as a dependent variable (Tables 5 and 6 in Appendix). The standardised regression coefficients are plotted in Fig. 2 which shows the actual path model. It can be seen that there are significant direct and indirect effects of gender and age on the number of working hours with employment position as the intermediate variable.

By focussing on *gender*, we can notice a direct standardised effect of − 0.199 on working hours. In addition to this, there are three indirect effects of gender on working hours when we include employment position and age as predictors:

I. The first indirect effect of gender can be seen through the employment position. This can be separated out into:

- A positive relationship of 0.053 between gender and employment position, as female GPs are more often salaried GPs than men
- A negative relationship between employment position and working hours of − 0.327, because salaried GPs work fewer hours than self-employed GPs (see also Table 1).

Hence, the indirect effect of gender by employment position on working hours is (0.053*− 0.327=) − 0.017.

II. The second indirect effect of gender on working hours can be seen through age. There is:

- An association between gender and age of − 0.204, because, on average, female GPs are younger than their male counterparts
- A positive effect of age on working hours of 0.097 as working hours increase when GPs are older.

Hence, the effect of gender on working hours by age is (− 0.204*0.097=) − 0.020.

III. Finally, there is a third indirect effect of gender on working hours by age and employment position. This results from:

- A negative effect of − 0.204 of gender on age, a negative relationship between age and employment position of − 0.448
- A negative relationship between employment position and working hours of − 0.327.

Thus, the third indirect effect of gender on working hours by age and employment position is (− 0.204*− 0.448*− 0.327=) − 0.030.

Taken together, the indirect effect of gender on working hours is − 0.067. This implies that 25.2% of the correlation (− 0.266) between both variables is explained by the abovementioned indirect effects. The largest part (74.8%) of the correlation is explained by the direct effect of gender on working hours.

Based on the path analysis and Fig. 1, a similar analysis can be performed focussing on age:

I. The first indirect effect of age on working hours seen through the employment job position is (− 0.448*− 0.327=) 0.146.
II. The second, through gender, is (− 0.204*− 0.199=) 0.041.
III. The third, through gender and employment position, is (− 0.204*0.053*− 0.327=) 0.004.

The sum of all indirect effects for age on working hours results in a standardised regression coefficient of 0.190, while this is 0.097 for the direct effect. Hence, the largest part of the correlation (0.288) between age and working hours can be explained by indirect effects (66.2%) and a smaller part by a direct effect (33.8%).

Table 4 summarises the indirect and direct effects of gender and age on working hours.

**Table 4:** Summary of direct and total indirect effects of gender and age on the number of working hours for GPs under the age of 60 (results are significant at 90% and 99% confidence level)

Effects on working hours:		Gender			Age	
		*β*/corr.	%		*β*/corr.^a^	%
Direct effect		− 0.199	74.8		0.097	33.8
Indirect effects		− 0.067	25.2		0.190	66.2
- via employment position	(0.053*–0.327)	− 0.017	6.5	(− 0.448*− 0.327)	0.146	50.8
- via age/gender	(− 0.204*0.097)	− 0.020	7.5	(− 0.204*− 0.199)	0.041	14.1
- via age/gender and employment position	(− 0.204*− 0.448*− 0.327)	− 0.030	11.2	(− 0.204*0.053*− 0.327)	0.004	1.2
Correlation coefficient		− 0.266	100.0		0.288	100.0

^a^The sum of the direct and indirect effects deviate from the correlation coefficient as a result of rounding up or down

## Discussion

### Summary of the results

The main question posed by this paper is how differences in the working hours of GPs can be explained by their gender, age, and employment position. If we know the relative and interacting impact of these variables upon the actual working hours of GPs, then this may help future workforce planning.

Based on bivariate analyses, we first found that female GPs work 8 h less than their male peers. GPs in their 50s worked the highest number of hours, followed by GPs age 60 and older. GPs younger than 40 worked the lowest number of hours. Multiple regression analysis showed that the relationship between working hours according to the age of GPs, for those GPs younger than 60, was not significantly different for women compared to men. In addition, by using a path analysis model, we found that a small part of the relationship between gender and working hours is explained by age and employment position, while the largest part (75%) is explained by a direct effect between both variables. This implies that female GPs consistently work fewer hours than their male counterparts, regardless of their age and employment position. Secondly, we found that the direct effect of age on working hours is relatively small (34%). Young GPs mainly work fewer hours compared to their older counterparts because these younger GPs are more often women and work as a salaried GP—that is in the service of another GP.

### Comparisons with other research

Our results are in line with many other studies from different countries showing that female physicians work fewer hours than their male counterparts [16, 22]. The results are also consistent with the finding that young physicians work fewer hours than their older peers [15]. This is shown for both male and female GPs below the age of 60. The lower number of working hours for young female GPs is often explained by their having children [9, 14, 16, 24]. Research into the domestic and family duties of physicians has shown that men are spending more hours on these activities when they have children, but this effect is at least twice as strong for women [25].

What has not been previously reported is that no significant differences exist between male and female GPs with regard to the relationship between age and working hours. This suggests that during their career and life course female and male GPs adapt their career to children similarly. However, our time use data does not contain information about the domestic arrangements of the GPs and whether they had children or not.

It is often cited that female GPs work fewer hours because they are generally younger and work more often on a salaried basis than male GPs [22]. Our study shows that the lower number of working hours for women compared to men is a consistent difference that barely changes when taking age and employment position into account. These results contradict the suggestion of van den Berg [26] that in the future gender would probably be irrelevant for differences in working hours. Van den Berg reported that the gender gap became smaller between 1987 and 2001; however, we found that in 2013 gender still appears to be the strongest determinant of GPs’ working time in the Netherlands. Therefore, we conclude that it is important to keep investigating the differences in working hours between the sexes, in particular from a policy perspective and in relation to health workforce planning.

As in many countries, the share of women in the GP workforce in the Netherlands has increased, and will increase further in the future, as most of the new entrants are women [9, 10]. This feminisation of the profession can put pressure on the availability of primary care [6, 13]. Policy makers responsible for health workforce planning are confronted with the challenge of sustaining a proper balance between the supply and the demand for care. It implies that more GPs need to be trained or recruited from other countries to meet this demand [27]. There are, however, other options for sustaining the level of services which have been suggested by some authors. A possible strategy is to develop family-friendly measures and flexible working conditions in order to keep women in the workforce [14, 28]. Another option is to organise the work more efficiently, for example by employing more support staff to perform standard tasks carried out by GPs [27]. Previous studies, however, have shown that the practice nurse (*praktijkondersteuner huisartsenzorg* or POH in Dutch) improved the quality of care, but did not yet reduce the workload for GPs [29, 30]. Further research is required to gain more insight into the value of different types of support staff and the most adequate skill mix in practices [31].

### Strengths and limitations

An important strength of this study is that it is based on a large dataset containing more than 61,000 time measurements during the working weeks of more than 1000 GPs. These measurements were obtained by a work sampling methodology using an SMS tool to monitor the activities of GPs each week in real time in a valid and user-friendly manner. Compared to the traditional time use survey methods, these unique data provide data of high quality to measure the working time of GPs.

Some limitations should be taken into account as well. Firstly, we studied the relevance of the life course by analysing the total and controlled differences between age categories. We speculate that the working hours of younger GPs may be lower related to childcare responsibilities and, conversely, higher at older ages when familial duties are less demanding. Our data, however, does not contain information on the age of respondents’ children. Therefore, we analysed only the working time difference according to the age of GPs and its interaction effect with gender. However, previous studies investigating the composition of households indicate that the presence of children is strongly related to the age of women or men [32]. A cohort analysis of women born in the 1940s, 1950s, and 1960s showed that approximately three quarters of the higher educated women, such as GPs, had children [33]. Secondly, this study analysed differences in working hours between individual GPs. Personal variables such as gender, age, and employment position explained a relatively considerable part (21%) of the variation in working hours, but 79% of this variance is still explained by other variables. These could, for example, concern the type of practice and the supporting staff. Considering the developments in the reallocation of tasks [34, 35], and the increasing number of co-owned practices in several countries [7, 36], future research is useful in order to gain more insight into the effects of these variables.

Finally, the analyses in this paper were based on cross-sectional data. The differences we found in working hours according to age, gender, and employment position provide no insight into how these can change over the course of time. Longitudinal data collected over several years can provide more insight into how the careers of female and male GPs develop during the course of their life with regard to their working hours.

## Conclusions

The proportion of female GPs is increasing, and they elect part-time employment more often, compared with their male peers. Furthermore, there is a new generation of female and male GPs who seem to choose to spend more time on leisure activities, and with their families, and therefore tend to work on salaried bases with which they can limit their working hours. These trends appear to suggest clear consequences for health workforce planning. The capacity of GP care may decline in the future. There is, however, still limited insight into the relative impact and interaction effects of gender, age, and employment position on working hours. Our analyses can be seen as a first step, presenting the important conclusion that the variation in working hours can be explained for a large part by the combination of age, gender, and employment position. Gender appears to be most important as a large part of the variation in working hours is explained by a direct effect of this variable and a smaller part by indirect effects.

## Appendix

**Table 5:** Effect of age and gender on employment position for GPs under the age of 60 (multiple regression)

Employment position	B	Beta	*p* value	
Intercept	1.246			
Gender (ref=male)	0.047	0.053	0.098	*
Age	− 0.023	− 0.448	0.000	***
Adjusted *R* square		21.1%	0.000	***

**p* < 0.1; ****p* < 0.01

**Table 6:** Effect of gender, age, and employment position on the number of working hours for GPs under the age of 60 (multiple regression)

Working hours	B	Beta	*p* value	
Intercept	43.810			
Gender (ref=male)	− 6.189	− 0.199	0.000	***
Age	0.173	0.097	0.007	***
Employment position (ref=self-employed)	− 11.324	− 0.327	0.000	***
Adjusted *R* square		20.9%	0.000	***

****p* < 0.01
